# Linking oxidative stress biomarkers to disease progression and antioxidant therapy in hypertension and diabetes mellitus

**DOI:** 10.3389/fmolb.2025.1611842

**Published:** 2025-05-26

**Authors:** Alberto J. Nuñez-Selles, Rodolfo A. Nuñez-Musa, Rafael A. Guillen-Marmolejos

**Affiliations:** ^1^ Research Division, Universidad Nacional “Pedro Henríquez Ureña” (UNPHU), Santo Domingo, Dominican Republic; ^2^ Contract Research Organization DR (CREODR), Santo Domingo, Dominican Republic; ^3^ Clínica “Corazones Unidos”, Santo Domingo, Dominican Republic

**Keywords:** oxidative stress, biomarkers, hypertension, diabetes, disease progression, antioxidant therapy, clinical trials, precision redox medicine

## Abstract

Oxidative stress (OS) is increasingly recognized as a key factor linking hypertension (HTN) and diabetes mellitus (DM). This review summarizes recent evidence regarding the dual role of OS as both an instigator and an amplifier of cardiometabolic dysfunction. In HTN, reactive oxygen species (ROS) produced by NADPH oxidases (NOXs) and mitochondrial dysfunction contribute to endothelial impairment and vascular remodeling. In DM, hyperglycemia-induced ROS production worsens beta-cell failure and insulin resistance through pathways such as the AGE-RAGE signaling, protein kinase C (PKC) activation, and the polyol pathway. Clinically validated biomarkers of OS, such as F2-isoprostanes (which indicate lipid peroxidation), 8-OHdG (which indicates DNA damage), and the activities of redox enzymes like superoxide dismutase (SOD) and glutathione peroxidase (GPx), show strong correlations with disease progression and end-organ complications. Despite promising preclinical results, the application of antioxidant therapies in clinical settings has faced challenges due to inconsistent outcomes, highlighting the need for targeted approaches. Emerging strategies include: 1. Mitochondria-targeted antioxidants to enhance vascular function in resistant HTN; 2. Nrf2 activators to restore redox balance in early diabetes; and 3. Specific inhibitors of NOX isoforms. We emphasize three transformative areas of research: (i) the interaction between the microbiome and ROS, where modifying gut microbiota can reduce systemic OS; (ii) the use of nanotechnology to deliver antioxidants directly to pancreatic islets or atherosclerotic plaques; and (iii) phenotype-specific diagnosis and therapy guided by redox biomarkers and genetic profiling (for example, KEAP1/NRF2 polymorphisms). Integrating these advances with lifestyle modifications, such as following a Mediterranean diet and exercising regularly, may provide additional benefits. This review outlines a mechanistic framework for targeting OS in the comorbidity of HTN and DM while identifying critical knowledge gaps, particularly regarding the timing of antioxidant signaling and the development of personalized redox medicine, which may serve as a reference for researchers and clinicians working in this area.

## 1 Introduction

Hypertension (HTN) and diabetes mellitus (DM) are interconnected metabolic disorders driving global cardiovascular morbidity, with oxidative stress (OS) implicated as a key pathological link (Myszko et al., 2025). OS arises from a disruption in redox homeostasis, where an overproduction of reactive oxygen species (ROS) overwhelms endogenous antioxidant defenses, leading to oxidative damage to lipids, proteins, and DNA (Sies et al., 2024). This imbalance accelerates endothelial dysfunction, insulin resistance, and end-organ damage, thereby perpetuating the progression of HTN and DM (Griendling et al., 2021; Yousef et al., 2023; Rais et al., 2024). Under physiological conditions, cells maintain redox homeostasis through tightly regulated systems, including enzymatic antioxidants (e.g., superoxide dismutase, SOD; catalase, CAT; glutathione peroxidase, GPx) and non-enzymatic scavengers (e.g., glutathione, vitamin E) (Lushchak, 2014). ROS, such as superoxide (O_2_•^−^) and hydrogen peroxide (H_2_O_2_), also serve as signaling molecules at low levels, modulating metabolic and vascular functions (Sies et al., 2020). However, in HTN and DM, chronic hyperglycemia, angiotensin II activation, and mitochondrial dysfunction exacerbate ROS production (Vejendla et al., 2024). The resulting oxidative overload disrupts redox-sensitive pathways (e.g., NF-κB, Nrf2), promoting inflammation, fibrosis, and cellular apoptosis (Fountoulakis et al., 2025).

Elevated levels of oxidative biomarkers—such as malondialdehyde (MDA), 8-hydroxy-2′-deoxyguanosine (8-OHdG), and F2-isoprostanes—reflect systemic OS and correlate with HTN severity and DM complications (Duni et al., 2017). For example, plasma MDA predicts endothelial dysfunction in HTN (Camargo et al., 2025), while urinary 8-OHdG is a surrogate for diabetic nephropathy (Spoto et al., 2025). Conversely, diminished antioxidant capacity (e.g., reduced SOD/GPx activity) is associated with β-cell failure and poor glycemic control (Newsholme et al., 2019). Despite their potential, biomarker variability and lack of standardized assays limit clinical adoption (Lankin et al., 2024). While preclinical studies demonstrate that antioxidants (e.g., vitamin E, N-acetylcysteine, polyphenols) mitigate OS and improve vascular function in HTN/DM (Black, 2022; He et al., 2023), human trials report inconsistent outcomes. Research indicates that high-dose vitamin E offers no cardiovascular benefits in individuals with DM (Kaye et al., 2025). In contrast, targeted therapies, such as mitochondria-specific antioxidants like MitoQ, show promising potential (Gioscia-Ryan et al., 2018). This disparity underscores the need for biomarker-guided personalized approaches. Consequently, the diagnosis of HTN and DM mediated by OS should consider an epigenetic perspective while factoring in the lifestyle of the target population.

This review evaluates: (i) the role of redox imbalance in HTN/DM progression; (ii) correlations between OS biomarkers and clinical outcomes; and (iii) current evidence on antioxidant adjuvant therapies. We aim to identify gaps in translating OS biomarkers into actionable therapeutic strategies by integrating mechanistic insights and clinical data. Additionally, the criteria emphasize the design of clinical trials for antioxidant therapies and potential mechanistic approaches for addressing HTN-DM in clinical practice.

## 2 Data search

**FIGURE 1:**
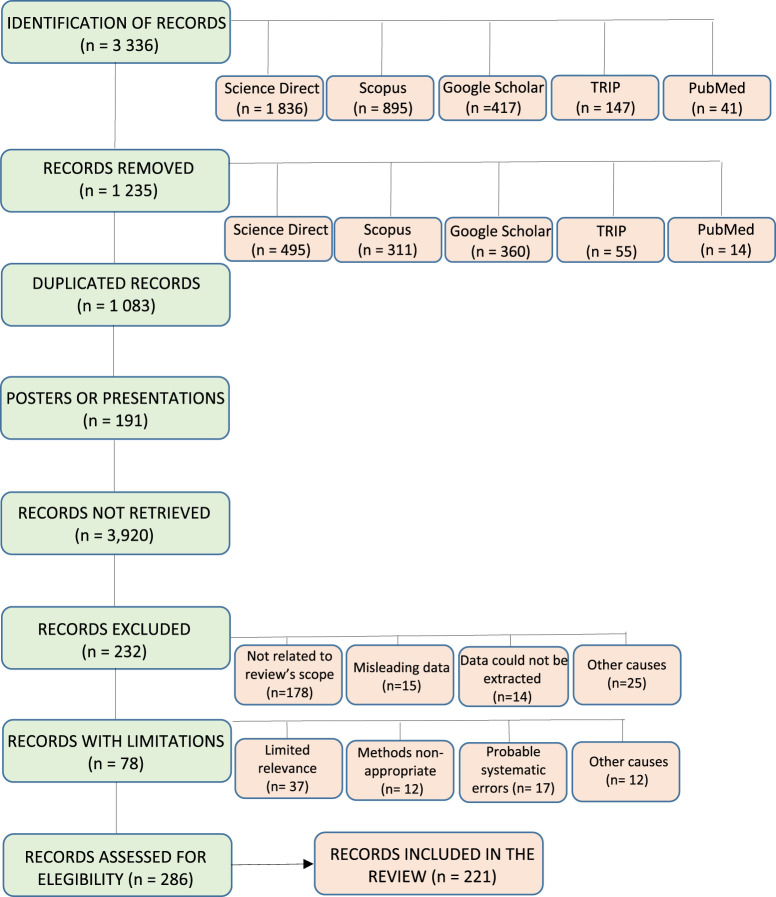
Flow chart diagram for the selection of records from databases.

An extensive search was done on published reports from specialized data sources, including PubMed/MedLine, ScienceDirect, Google Scholar, SciFinder, Scopus, and the TRIP Database. The search methodology included terms as oxidative stress, reactive oxygen species, biomarkers, hypertension, endothelial dysfunction, mitochondrial dysfunction, diabetes, β-cell damage, vascular dysfunction, hyperglycemia, insulin resistance, vascular complications, diabetic neuropathies, disease progression, antioxidant therapy, clinical trials, and precision redox medicine. The search included *in vitro*, *in vivo*, and clinical studies published between 2014 and 2025. These studies are expected to provide comprehensive data on the relationship between ROS and lifestyle, as well as the metabolic pathways involved in HTN and DM. Published reports were downloaded and reviewed from specialized data sources. The number of records related to HTN and DM in Science Direct rose significantly from 1,453 in 2014 to 4,003 in 2024, indicating a growing interest in this topic within the scientific community. After refining the data across all databases, 3,336 records were registered. Out of these, 221 were included in the review, as shown in Figure 1.

## 3 Current diagnosis of oxidative stress

The most common approach to diagnose OS is the static measurement of OS biomarkers, but the effective management of OS relies on assessing oxidative burden accurately and implementing targeted therapies that extend beyond mere ROS scavenging. Direct and indirect biomarkers are used as follows:

• Lipid peroxidation: Malondialdehyde (MDA) is measured via the Thiobarbituric Acid-Reactive Substances (TBARS) assay, while F_2_-isoprostanes (stable end-products of arachidonic acid oxidation) are quantified by gas or liquid chromatography-mass–mass spectrometry (HPLC/MS-MS) for high specificity (Sabitha et al., 2024).
• Protein oxidation: Protein carbonyl groups serve as reliable biomarkers of protein oxidation. The most widely used detection method involves the derivatization with 2,4-dinitrophenylhydrazine (DNPH) and spectrophotometric quantification at 370–375 nm, with the absorbance being proportional to carbonyl content. Alternatively, immunoblotting with anti-DNP antibodies and ELISA-based approaches for higher throughput are applied (Kehm et al., 2021).
• DNA oxidation: 8-hydroxy-2′-deoxyguanosine (8-OHdG) is a well-established biomarker of OS for DNA oxidation, reflecting guanine base damage caused by ROS. Its levels are commonly quantified using high-performance liquid chromatography (HPLC) coupled with electrochemical or mass spectrometry detection (HPLC-ECD or HPLC-MS/MS), offering high sensitivity and specificity. ELISA kits provide a cost-effective and high-throughput alternative, though potential antibody cross-reactivity requires careful validation. Elevated 8-OHdG levels correlate with aging, cancer, diabetes, and neurodegenerative diseases, making it a critical tool for assessing oxidative DNA damage in clinical and experimental research (Chiorcea-Paquim, 2022).
• Electron Paramagnetic Resonance Spectroscopy (EPR): It is a powerful tool to directly detect and quantify ROS in biological tissues. EPR spin trapping and fluorescent probes such as DCFH-DA enable dynamic monitoring of intracellular ROS (Ioannidou et al., 2025). EPR has also identified elevated superoxide anion (O_2_•^−^) in glomeruli and tubules of diabetic rodents, correlating with albuminuria. (Epel et al., 2022). Moreover, vascular ROS (e.g., peroxynitrite) in resistant HTN models are quantified using NOX-specific probes. (Babić and Peyrot, 2019; Valaitienė and Laučytė-Cibulskienė, 2024).
• Omics Approaches: Redox proteomics and transcriptomics identify oxidative modifications and gene expression patterns, aiding personalized therapy (Dai and Shen, 2022)

All these assays, both traditional and advanced, single or combined, provide a comprehensive picture of oxidative damage across biomolecular targets. However, dynamic measurement of the OS state and progression has been developed recently, which will be discussed ahead with the focus on HTN and DM.

## 4 The role of lifestyle and the need for antioxidant supplementation in hypertension and diabetes

The interplay between lifestyle factors and OS is pivotal in the development and progression of HTN and DM. Modern sedentary behaviors, poor dietary habits, smoking, and chronic psychological stress exacerbate ROS production while impairing antioxidant defenses (Pizzino et al., 2017). Conversely, lifestyle modifications, including physical activity, Mediterranean or DASH (Dietary Approaches to Stop Hypertension) diets, smoking cessation, and stress management, have been shown to restore redox balance and reduce disease severity (Fogacci et al., 2019). The Mediterranean diet, rich in polyphenols (e.g., olive oil, nuts, berries), reduces lipid peroxidation (MDA) and enhances antioxidant enzymes (SOD, GPx) in diabetic patients (Ruiz-Pozo et al., 2024). The DASH diet reduces blood pressure (BP) and OS markers by enhancing nitric oxide (NO) bioavailability and decreasing NADPH oxidase (NOX) activity (Muszalska et al., 2025).

Moderate aerobic exercise upregulates endogenous antioxidants (e.g., SOD, CAT) and improves mitochondrial function in HTN and DM (Jiang et al., 2023). However, excessive exercise without proper recovery can paradoxically increase ROS, highlighting the need for balanced regimens (Margaritelis et al., 2018). Chronic sleep deprivation and stress elevate cortisol and pro-oxidant cytokines, worsening insulin resistance and endothelial dysfunction (Aboonabi and McCauley, 2024). Mindfulness and yoga have been shown to reduce OS biomarkers (e.g., F2-isoprostanes) in hypertensive patients (Lee et al., 2020). Considering these factors, is antioxidant supplementation necessary for those with HTN and DM?

The role of exogenous antioxidant supplementation remains controversial since meta-analyses have shown mixed results. Unlike endogenous antioxidants, synthetic supplements, such as vitamin C pills, may disrupt redox signaling by non-specifically scavenging ROS, including beneficial low-level oxidants that play a role in metabolic regulation (Forman and Zhang, 2023). Some targeted antioxidant supplementation has shown positive results, like vitamin D and Omega-3 Fatty Acids (OFAs), which have been promising in reducing inflammation and OS in HTN/DM when deficiency is confirmed (Mazidi et al., 2020). Recent studies suggested that fish oil may have beneficial effects in treating HTN, primarily due to various bioactive oxidation products formed by free radicals from OFAs. However, the specific molecular species responsible for these effects remains unidentified (Krupa et al., 2024). OFAs are linked to a reduced incidence of cardiovascular diseases. Evidence from randomized controlled trials strongly supports the BP-lowering effects of fish oil, which is rich in eicosapentanoic acid (EPA) and docosahexanoic acid (DHA) (Engler, 2017). Research has examined the impact of several doses of OFAs on HTN in clinical trials, ranging from 2 to 4 g per day. A meta-analysis of 36 randomized clinical trials found that supplementation with fish oil, providing an average of 3.7 g per day of EPA and DHA, resulted in a reduction of systolic BP (SBP) by 2.1 mm Hg and diastolic BP (DBP) by 1.6 mm Hg. (Miller et al., 2014). A recent review has connected various clinical events to redox factors and OS in cardiovascular pathophysiology, providing evidence for new pharmacological therapies such as OFAs, non-selective beta-blockers, and microRNAs (Farías et al., 2017). Polyphenol-rich extracts (e.g., curcumin, resveratrol) improve endothelial function in early-stage diabetes, likely due to their pleiotropic effects beyond mere ROS scavenging (Mohd-Nor et al., 2022). N-acetylcysteine (NAC) may benefit patients with severe oxidative damage (e.g., diabetic nephropathy) by replenishing glutathione (Dróżdż et al., 2023).

Given the variability in individual redox status, future strategies should consider measuring OS biomarkers (e.g., MDA, SOD, GPx) to identify patients who may benefit from supplementation; combining antioxidants with lifestyle changes (e.g., Mediterranean diet + CoQ10) for synergistic effects (González-Guardia et al., 2015); and avoiding blanket supplementation in patients with adequate redox balance to prevent interference with physiological ROS signaling (Sies et al., 2024; Nuñez-Sellés et al., 2019).

While lifestyle modifications remain the cornerstone of redox balance restoration in HTN and DM, targeted antioxidant supplementation may be justified in selected cases. Future research should focus on personalized antioxidant therapy guided by biomarker profiling to optimize clinical outcomes.

## 5 Oxidative stress and hypertension

**FIGURE 2:**
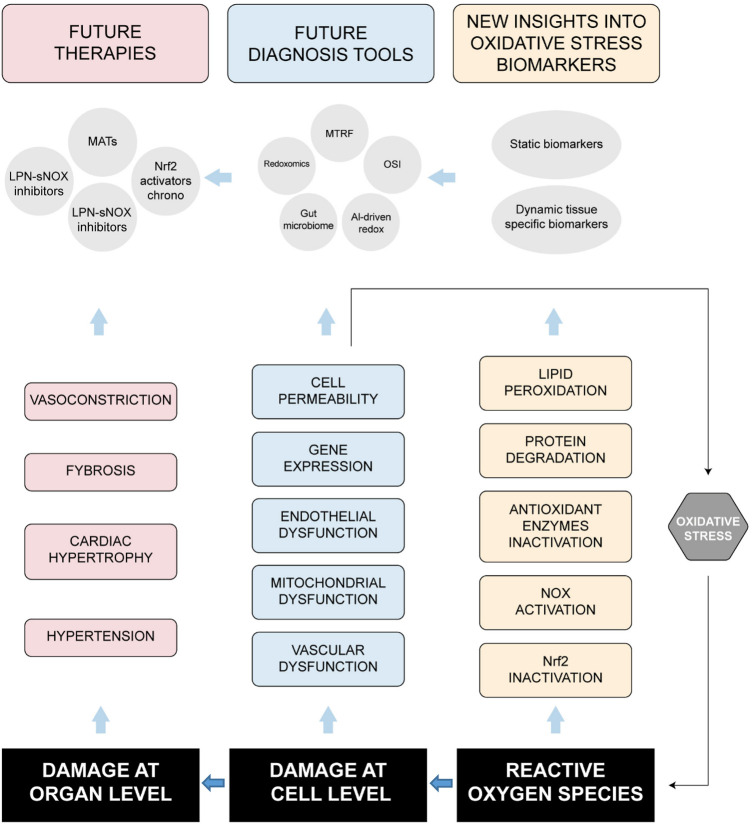
Linking oxidative stress with cell and organ damages to cardiovascular dysfunctions leading to hypertension progression.

Recent reviews have examined OS and the sources of ROS concerning HTN (Kreutzmann et al., 2023; Fountoulakis et al., 2025) as well as antioxidant therapeutic approaches (Młynarska et al., 2024; Kumar et al., 2023; Pari et al., 2024). Several *in vitro* and *in vivo* studies suggest that ROS activate specific molecular mechanisms that ultimately lead to elevated BP levels (Tain and Hsu, 2022). The role of OS in HTN remains a “chicken-or-egg” debate. Preclinical studies have shown a genetic deletion of antioxidant enzymes (e.g., SOD, GPx) inducing HTN in mice (Carlström et al., 2010), whereas human data led to elevated OS biomarkers (e.g., F2-isoprostanes) preceding HTN onset in normotensive individuals (Rodrigo et al., 2021). These findings suggest that OS may be a cause of HTN. However, there is also evidence indicating that OS can be a consequence of HTN, particularly due to mechanical stress from high BP activating reactive ROS-producing enzymes, such as NOXs (Camargo et al., 2025). Additionally, antioxidants like vitamin C have been shown to lower BP in individuals with pre-HTN, but they do not have the same effect in those with established HTN (Camargo et al., 2025). Therefore, OS is both a trigger and an amplifier of HTN, which creates a vicious cycle. A summary of how ROS are related to HTN at the cell and organ levels is shown in Figure 2.

Clinical studies have reported controversial outcomes despite support from basic research and pre-clinical studies (Table 1). This may be due to the complex pathophysiological nature of ROS signaling in humans with comorbidities. (Egea et al., 2017). The importance of antioxidant therapy in managing OS-induced HTN has been emphasized in a review of various preclinical studies and clinical trials (Amponsah-Offeh et al., 2023; Tenório et al., 2018). These studies highlight the significance of using antioxidants in HTN management (Ahmad et al., 2017). ROS are produced during normal cellular metabolic processes, while antioxidants work to eliminate these oxidants when they are in excess and repair the damage caused by ROS (Vakifahmetoglu-Norberg et al., 2017). Under normal physiological conditions, intracellular OS results from the abnormal production of ROS during typical mitochondrial respiration. It can also occur during the reperfusion of hypoxic tissue and concerning infection and inflammation (Sekhon et al., 2017). The excessive production of ROS has been associated with endothelial injury and contributes to both extracellular and intracellular OS (Incalza et al., 2017).

**TABLE 1:** Clinical trials of antioxidant therapies in hypertension (2014–2024).

Intervention	Trial	Population	Key findings	Limitations	References
Coenzyme Q10	Q-SYMBIO	Chronic HF + HTN (n = 420)	↓ SBP by 11 mmHg (p < 0.01)	Small sample size	Mortensen et al. (2014)
Vitamin C + E	HOPE-TOO	High-risk CVD (n = 7,030)	No reduction in BP or CVD events	Non-targeted approach. Broad spectrum	Młynarska et al. (2024)
Resveratrol	Meta-analysis	HTN (n = 407)	No reduction in BP or CVD events	No long-term follow-up	Fogacci et al. (2019)
Allopurinol	CKD-FIX	HTN + CKD (n = 369)	No BP change despite ↓ uric acid	Off-target effects	Badve et al. (2020)
Omega-3 FAs	STRENGTH	HTN + Hypertriglyceridemia	No BP benefit (4 g/day)	Confounded by lipid effects	Nicholls et al. (2020)
Melatonin	MEL-HT	Nocturnal HTN (n = 45)	↓ Nighttime SBP by 7 mmHg (p = 0.01)	Small, single-center trial	Ramos-Gonzalez et al. (2023)
N-Acetylcysteine	NAC-HYP	HTN-II (n = 120)	↓ MDA No BP change	Lack of hard endpoints	Gutziet et al. (2021)
MitoQ	MITO-HTN	Resistant HTN (n = 60)	Improved FMD ↑ 2.1% (p = 0.03)	Short duration (8 weeks)	Pang et al. (2025)
Sulforaphane	SFN-HTN	Diabetic HTN (n = 90)	↓ DBP by 5 mmHg ↑ Nrf2 activity (p < 0.05)	Biomarker-only focus	Mangla et al. (2021)
Polyphenol-rich olive oil	PREDIMED-Plus	Metabolic syndrome (n = 6,874)	↓ SBP by 3.2 mmHg (MedDiet + EVOO, p = 0.02)	Lifestyle co-interventions	Rosello et al. (2024)

HTN: hypertension, BP: blood pressure, SBP: systolic blood pressure, DBP: diastolic blood pressure, FMD: Flow-Mediated Dilation, CVD: cardiovascular, CKD: chronic kidney disease, MDA: Malonyl dialdehyde.

The efficacy of antioxidant therapy in HTN depends on the HTN degree (I, II, or III). It has been demonstrated that OS was not present in HTN-I patients, and significantly, it was lower as compared to a healthy control group in an observational study with 355 subjects (Nuñez-Sellés et al., 2019). However, OS was moderate or severe for HTN-II and HTN-III, respectively. Most of the HTN-I patients had a combination treatment (ACE inhibitor + statin), which has been reported to have antioxidant effects (Profumo et al., 2014). In mild HTN, endogenous antioxidant enzymes were not decreased, and thus antioxidant therapy in early stages of HTN should not be a critical issue. Nevertheless, antioxidant supplementation would be recommended for HTN-II and HTN-III patients.

Certain medications used to treat HTN, such as ACE inhibitors and angiotensin (Ag II) receptor AT1 antagonists, have shown antioxidant properties. These antioxidant effects may partially contribute to the overall benefits that these drugs provide in managing HTN. Ang II activates AT1 receptors, which can lead to various effects on vascular cells, including vasoconstriction, cell growth, and the induction of pro-inflammatory cytokines, as well as pro-fibrogenic actions (Silva-Velasco et al., 2024). Furthermore, Ang II stimulates the production of ROS within cells by activating NOXs. The production of ROS induced by Ang II is crucial in the development of HTN (Masi et al., 2019). As a result, Ang II is regarded as one of the most significant triggers of OS, both locally and systemically. Therefore, it is not surprising that both ACE inhibitors and AT1 antagonists are effective in reducing OS on various cardiovascular diseases (Ajoolabady et al., 2024).

### 5.1 Endothelial dysfunction

Endothelial dysfunction and OS are inextricably linked in the pathophysiology of HTN, leading to vascular damage and elevated BP. The vascular endothelium, a critical regulator of vascular tone, becomes dysfunctional when excessive ROS impairs nitric oxide (NO) bioavailability, a hallmark of HTN. Key sources of ROS include NOXs activation, uncoupled endothelial nitric oxide synthase (eNOS), and mitochondrial dysfunction, all of which are exacerbated by hypertensive stimuli such as Ang II and mechanical shear stress. When ROS (e.g., O_2_•^−^) scavenges NO, vasodilation is compromised, leading to increased peripheral resistance. Additionally, ROS promotes pro-inflammatory signaling (e.g., NF-κB activation) and endothelial adhesion molecule expression (e.g., VCAM-1, ICAM-1, TNF-α), further exacerbating vascular stiffness and atherosclerosis (Zeng and Yang, 2024). OS also reduces tetrahydrobiopterin (BH4), a critical cofactor for eNOS, causing it to produce more ROS instead of NO, thereby amplifying endothelial injury (Hernandez-Navarro et al., 2024). Clinically, this manifests as impaired flow-mediated dilation (FMD) and elevated biomarkers like asymmetric dimethylarginine (ADMA), an endogenous eNOS inhibitor (Kavurma et al., 2022). Therapeutic strategies targeting this axis (Figure 2), such as BH4 supplementation, NOX inhibitors, and lifestyle modifications (e.g., exercise, Mediterranean diet), aim to restore redox balance and endothelial function, though their efficacy varies by HTN stage and comorbidities. Thus, endothelial OS is both a driver and consequence of HTN, making it a pivotal target for interventions aimed at breaking the cycle of vascular dysfunction.

### 5.2 Mitochondrial dysfunction

Mitochondrial dysfunction plays a pivotal role in the pathogenesis of HTN-related OS, acting as both a major source and a vulnerable target of ROS in the cardiovascular system. In hypertensive states, mitochondria generate excessive ROS due to electron transport chain (ETC) disruption, particularly at complexes I and III, coupled with reduced antioxidant defenses (e.g., Mn-SOD) (Grossini et al., 2025). This oxidative burden is exacerbated by Ang II, which promotes mitochondrial fission via dynamin-related protein 1 (Drp1) and inhibits mitophagy, leading to the accumulation of damaged, ROS-producing mitochondria (Tábara et al., 2025). Concurrently, mitochondrial ROS (mtROS) amplifies vascular dysfunction by oxidizing BH4, uncoupling endothelial eNOS, and reducing NO bioavailability while also activating pro-inflammatory pathways (e.g., NLRP3 inflammasome) and stimulating NOX activity, creating continuous oxidative damage (Penna and Pagliaro, 2025). mtROS further contributes to vascular remodeling by inducing smooth muscle cell proliferation and endothelial apoptosis via oxidative DNA damage (8-OHdG) and lipid peroxidation (MDA). Importantly, mitochondrial DNA (mtDNA) mutations, which accumulate with age and HTN, perpetuate this dysfunction, rendering the vasculature more susceptible to oxidative injury. Therapeutic strategies targeting mitochondrial OS, known as mitochondrial-targeted antioxidants (MTAs) such as MitoQ, are modulators and promoters of mitochondrial biogenesis (PGC-1α activators), which have shown promise in preclinical HTN models (Halling et al., 2019).

Within the therapeutic tools proven effective, Elamipretide (SS-31) is one of the most promising. Elamipretide is a cell-permeable tetrapeptide that selectively accumulates in mitochondria, binds cardiolipin, stabilizes inner-membrane structure, inhibits excessive ROS production, and preserves ATP synthesis. In preclinical heart-failure models, elamipretide improved left ventricular function and attenuated remodeling. (Tung et al., 2025). Animal studies demonstrated cognitive protection and reduced inflammation in models of chronic sleep deprivation and DM2 (Zhang et al., 2024). Early clinical studies in Barth syndrome, primary mitochondrial myopathies, and age-related macular degeneration have shown favorable safety, tolerability, and signals of efficacy (Thompson et al., 2024). So far, clinical trial data for elamipretide and bardoxolone underscore the viability of mechanism-driven therapies in treating OS–related diseases (Karaa et al., 2024). These results justify ongoing Phase III trials exploring elamipretide’s therapeutic potential.

Evidence about the role of mtROS has led to the development of MATs for therapeutic intervention in several diseases, including HTN -Figure 2 (Jiang et al., 2020; Colon-Hidalgo et al., 2022). MitoQ has shown promising preventive benefits for several metabolic disorders, including peripheral arterial HTN (Pool et al., 2021), whereas MitoTEMPO can scavenge mtROS, reduce oxidative damage to mitochondria, restore mitochondrial function, and help maintain normal cellular function (Chang et al., 2024). Clinical translation for using MTAs requires addressing challenges like tissue-specific delivery and long-term safety. Thus, mitochondrial dysfunction represents a critical hub in HTN-associated OS, linking metabolic, inflammatory, and vascular pathways, and its modulation offers a novel avenue for precision therapies in resistant HTN.

### 5.3 NADPH oxidases (NOXs)

NOXs are the only known enzymes specifically responsible for generating ROS (Bedard and Krause, 2007). There are seven human NOX enzymes identified: NOX1, NOX2, and NOX3, which are regulated by various cellular proteins; NOX5 and DUOXs, which are activated by calcium due to additional calcium-binding domains; and NOX4, the only constitutively active member of this family (Chocry and Leloup, 2020). NOXs play a crucial role in coordinating cell damage, stress responses, and tissue regeneration. High levels of ROS, resulting from the hyperactivity of NOXs, can lead to genetic instability. This instability can cause excessive cellular proliferation, activation of the DNA damage response, proliferative senescence, and apoptosis (Ogrunc et al., 2014). Ang II plays a crucial role in the progression of HTN and is one of the most significant regulators of NOX activity in vascular cells. Consequently, researchers have extensively investigated the role of these enzymes in Ang II-dependent HTN (Pinheiro and Oliveira-Paula, 2020). Developing selective NOX inhibitors has been quite challenging due to the high degree of similarity in the core catalytic domains of these enzymes, which makes it difficult to achieve selectivity for specific isoforms. Current research trends in NOX inhibitors focus on creating compounds that are both highly effective and specific to individual isoforms, as shown in Figure 2 (Dustin et al., 2023).

### 5.4 The nuclear factor erythroid 2-related factor 2 (Nrf2)

The Nrf2 pathway serves as a critical endogenous defense mechanism against OS in HTN, orchestrating the transcription of antioxidant and cytoprotective genes that counteract vascular dysfunction (Lal et al., 2024). Under physiological conditions, Nrf2 remains sequestered in the cytoplasm by its inhibitor, the Kelch-like ETC-associated protein 1 (Keap1), but oxidative or electrophilic stress triggers Nrf2 dissociation, nuclear translocation, and binding to antioxidant response elements (AREs), upregulating genes such as heme oxygenase-1 (HO-1), NAD(P)H quinone oxidoreductase 1 (NQO1), and glutathione S-transferases (GSTs) (Cuadrado et al., 2019). In HTN, chronic activation of Ang II and NOXs overwhelms this system, leading to Nrf2 dysfunction or exhaustion, which exacerbates ROS accumulation, endothelial dysfunction, and vascular remodeling. Sulforaphane from cruciferous vegetables (e.g., broccoli), bardoxolone methyl, and dimethyl fumarate have demonstrated promise in preclinical models by restoring redox balance, reducing BP, and attenuating end-organ damage effects attributed to enhanced HO-1-mediated vasodilation, reduced NOX activity, and suppression of pro-inflammatory NF-κB signaling (Alves et al., 2025; Simões e Silva, 2025; Jindam et al., 2024).

Unlike direct antioxidants, Nrf2 activators amplify endogenous defenses, offering sustained protection. The pivotal role of the Nrf2-Keap1 signaling pathway in maintaining redox, metabolic, and protein homeostasis, as well as regulating inflammation responses, has been discussed (Dinkova-Kostova and Copple, 2023). It was highlighted that inducible activation of Nrf2 promotes cytoprotective mechanisms against various immune, neurodegenerative, and metabolic disorders characterized by OS and inflammation. Novel prodrugs of monomethyl fumarate have achieved site-specific release at OS loci via Baeyer–Villiger oxidation, minimizing systemic side effects while activating Nrf2 locally (Avery et al., 2024).

However, the therapeutic potential of Nrf2 activation is tempered by paradoxical risks; prolonged overactivation may promote oncogenic pathways or, as seen in clinical trials with bardoxolone methyl, adverse renal outcomes (Pergola et al., 2021). Genetic polymorphisms in Keap1 or Nrf2 further influence individual susceptibility to HTN and response to therapy, highlighting the need for personalized approaches (Begum and Lakshmanan, 2023). While the Nrf2 pathway represents a compelling target for mitigating OS in HTN, its dual-edged nature requires careful modulation to harness its protective effects without triggering unintended consequences.

## 6 Oxidative stress and diabetes mellitus

OS in DM operates as both a causal driver and consequential amplifier of disease progression, accelerating metabolic dysfunction and tissue damage in a similar way as in HTN (Caturano et al., 2025). Chronic hyperglycemia fuels excessive ROS production through multiple pathways, including mitochondrial ETC leakage, NOX activation, and advanced glycation end products (AGE)-mediated RAGE signaling. These ROS directly impair pancreatic β-cell function by suppressing insulin gene expression (via PDX-1 inactivation) and triggering apoptosis through JNK and NF-κB pathways, thereby reducing insulin secretion (Kaneto et al., 2022). Simultaneously, ROS disrupts insulin signaling in peripheral tissues by promoting IRS-1 serine phosphorylation and degrading phosphatidylinositol-3-kinase (PI3K)/Akt activation, leading to insulin resistance (Papaetis et al., 2025). Conversely, the resulting hyperglycemia and dyslipidemia further exacerbate OS through four key mechanisms: (1) the polyol pathway, where glucose conversion to sorbitol depletes NADPH and glutathione; (2) AGE-RAGE interactions that activate pro-inflammatory cascades and additional ROS generation; (3) protein kinase C (PKC) activation, which upregulates NOX isoforms; and (4) lipid peroxidation, where free fatty acids (FFAs) overload mitochondrial β-oxidation, generating cytotoxic aldehydes like 4-hydroxynonenal (4-HNE) (Młynarska et al., 2024). This bidirectional relationship is clinically evident in the early elevation of OS biomarkers (e.g., urinary 8-OHdG, serum F2-isoprostanes) preceding overt DM and their correlation with disease severity and complications (e.g., retinopathy, nephropathy). Therapeutic strategies targeting this cycle -such as Nrf2 activators (sulforaphane), mitochondrial antioxidants (MitoQ), and AGE inhibitors (aminoguanidine)- highlight the dual role of OS as interventions early in DM, which can improve β-cell function, while later-stage treatments primarily mitigate complications. Thus, OS in DM represents a paradigm of metabolic memory, where initial oxidative damage perpetuates dysfunction long after glycemic control is achieved, underscoring the need for early redox-targeted therapies.

Some researchers suggest that acute spikes in plasma glucose trigger this stress (Papachristoforou et al., 2020), while others attribute it to glycemic variability (Rehman and Akash, 2017). If antioxidant levels are adequate, oxidative damage to key biomolecules in type 2 DM (DM2) may be minimized, potentially leading to improved outcomes. This has led to the proposal of investigating antioxidant supplementation as an adjunct therapy to reduce oxidative stress and help to prevent vascular complications (Nuñez-Sellés et al., 2019; Choi and Ho, 2018).

**FIGURE 3:**
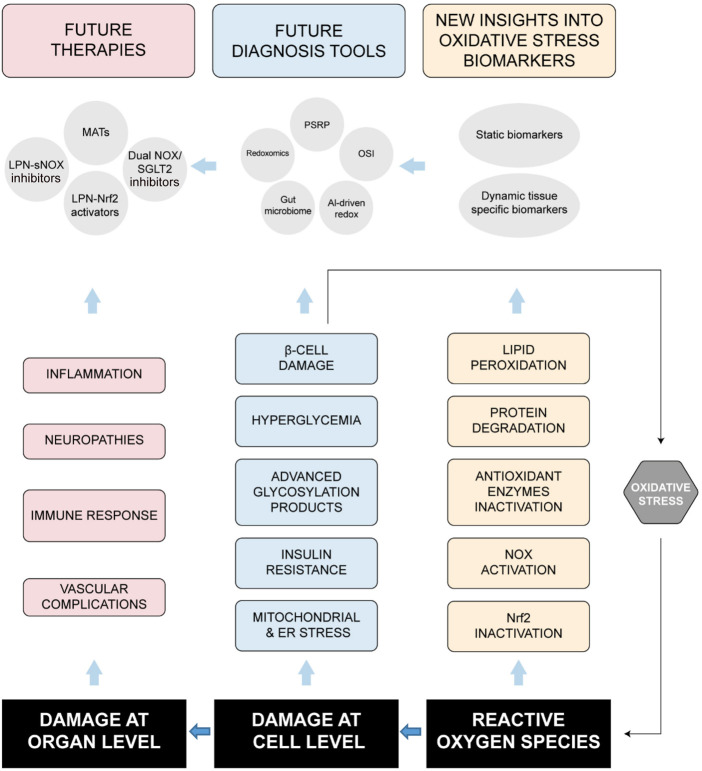
Linking oxidative stress with cell and organ damages to cardiovascular dysfunctions leading to diabetes mellitus progression and complications.

The effective management of blood glucose levels in individuals with DM2 relies on an integrative approach. This approach includes patient education, lifestyle modifications, pharmacotherapy, and vigilant monitoring for potential complications (Garber et al., 2021). Despite these guidelines, the burden of DM2 remains significant, posing considerable challenges for affected individuals and straining the healthcare system. As a result, developing new therapeutic strategies for preventing and managing DM2 continues to be a key area of research. One particular focus is the role of OS and inflammation. Research has shown that chronic hyperglycemia leads to OS and inflammation, which contribute to the onset and progression of DM2, as well as its associated complications, including renal and cardiovascular diseases (Pickering et al., 2018). One of the primary research challenges is reducing OS to alleviate diabetes symptoms. However, the insufficient evidence supporting the beneficial effects of antioxidants in preventing OS-related diseases has prompted the development of new strategies. One promising avenue involves the use of new inhibitors that target major ROS-producing systems, such as HO-1 expression. This approach offers an alternative to traditional antioxidant therapies for treating endothelial dysfunction (Arad et al., 2020). As a result, Nrf2 increases the expression of various well-known antioxidant markers, such as glutathione, HO-1, SOD, and NADPH quinone reductase (Paunkov et al., 2019). A summary of how ROS are related to DM is shown in Figure 3.

Clinical studies have reported controversial results despite the support from preclinical studies (Table 2). Several gene-therapy strategies aimed at countering OS in DM2. In an extended review, it was highlighted that some preclinical gene-therapy approaches delivering exogenous antioxidant enzymes, via viral vectors or nanozyme platforms, in animal models of hyperglycemic bone defects, significantly reduced inflammation, limited ROS-mediated damage, and improved healing outcomes (Li et al., 2024). A special gene therapy approach, that delivers treatment directly to heart and blood vessel cells, uses harmless viruses called AAVs (Adeno-Associated Viruses) as delivery vehicles to carry therapeutic genes into target cells (Weeks and Bernardo, 2025). Current research focuses on improving delivery precision to specific cell types, enhancing treatment effectiveness, and ensuring safety for clinical use. One main aspect is the choice of AAV serotype and capsid engineering to reach natural tropism. Serotypes such as AAV1, AAV6, AAV8, and especially AAV9 have demonstrated efficient cardiac transduction in small and large animals, enabling robust gene transfer to myocardium with intracoronary or intravenous delivery (Mazurek et al., 2024).

**TABLE 2:** Clinical trials of antioxidant therapies in diabetes mellitus (2014–2024).

Therapy	Trial	Population	Key outcomes	Limitations	References
Alpha-Lipoic Acid	ALA/Neuropathy	DM2 with neuropathy (n = 72)	Reduced pain and disability scores by more than 30% (p < 0.01)	Small sample size	Agathos et al. (2018)
MitoQ (Mitochondrial-targeted)	MitoQ	DM2 (n = 169)	Protective role on vasculature (inflammation markers and prevention of neuropathy	Small sample size	Escribano-Lopez et al. (2016)
Sulforaphane (Nrf2 activator)	SFN-DM2	DM2 (n = 97)	↓ HbA1c by 0.5% (p = 0.04); ↑ Nrf2 activity	Short duration (12 weeks)	Axxelson et al. (2017)
Coenzyme Q10	CoQ10-DPP4	DM2 (+metformin) (n = 120)	Improved insulin sensitivity (HOMA-IR ↓18%, p < 0.05)	No CV outcome data	Dludla et al. (2020)
Bardoxolone Methyl	TSUBAKI	Diabetic CKD (n = 121)	eGFR ↑5.1 mL/min (p < 0.01); albuminuria ↓32%	Discontinued due to safety concerns	Pergola et al. (2021)
Resveratrol	RESDIAB	Prediabetes (n = 75)	↓ Fasting glucose by 0.8 mmol/L (p = 0.02)	No long-term follow-up	Sattarinezhad et al. (2019)

DM2: type 2 diabetes, ALA: alpha-lipoic acid, Nrf2: Nuclear Factor Erythroid 2-Related Factor 2, CKD: chronic kidney disease, HbAc1: Glycosylated Haemoglobin, HOMA-IR: homeostasis model assessment of insulin resistance.

Another approach is the management of calcium-handling proteins, which have had variable results (Khan et al., 2025). A cardiac-bridging integrator 1 (cBIN1) delivered with AAV9 reversed chronic ischemic heart failure in canine models, improving survival and contractility. S100A1, another calcium-binding protein, has regulated cardiac contractility and calcium-handling. Its downregulation is linked to heart failure and hypertrophy. Anti-remodeling targets acting over regulating genes of the S100A1, like phospholamban-targeted shRNA, and antioxidant enzymes (e.g., CAT) are under preclinical evaluation for preventing pathological hypertrophy and fibrosis. A study highlighted S100A1’s ability to restore calcium cycling in cardiomyocytes, improving contractility in heart failure models, and gene therapy using AAV-S100A1 showed reduced fibrosis and hypertrophy in rodents (Rostami et al., 2025).

### 6.1 β-Cell damage by ROS

Pancreatic β-cells exhibit a unique vulnerability to OS due to their inherently low expression of antioxidant enzymes (SOD, CAT, and GPx) and their high metabolic activity, which generates substantial ROS under hyperglycemic conditions (Eguchi et al., 2021). Several conditions leading to ROS generation in *β* cells have been proposed, among which are hyperglycemia, hyperlipidemia, hypoxia, and endoplasmic reticulum (ER). Hyperglycemia, the defining diabetes, can be directly associated with increased ROS generation through a variety of mechanisms (Singh et al., 2025).

Chronic hyperglycemia drives mitochondrial overstimulation, leading to excessive electron leakage at complexes I and III of the ETC, producing superoxide anions (O_2_•^-^). These ROS trigger a cascade of detrimental effects: (1) transcriptional suppression of insulin via oxidative inactivation of PDX-1 and MafA, key regulators of insulin gene expression (Guo et al., 2023); (2) ER stress, where unfolded protein response (UPR) pathways (PERK, IRE1α, ATF6) shift from pro-survival to pro-apoptotic signaling (Eizirik et al., 2020); and (3) direct DNA damage, evidenced by elevated 8-OHdG in diabetic islets (Duni et al., 2017). Additionally, ROS activates stress-sensitive kinases (JNK, p38 MAPK) that phosphorylate IRS-2, accelerating its proteasomal degradation and further impairing β-cell survival (Liu et al., 2020). The resulting β-cell dysfunction manifests clinically as impaired glucose-stimulated insulin secretion (GSIS) and, ultimately, apoptosis—a process confirmed in human autopsies showing ca. 40% reduced β-cell mass in DM2 (Maheshvare et al., 2023).

Recent single-cell RNA sequencing has identified a subset of ROS-resilient β-cells that upregulate SIRT3 and FOXO1, suggesting potential therapeutic targets for preservation (Nie et al., 2024). Additionally, it has been reported that OS affects epigenetic regulatory mechanisms involved in the regulation of pancreatic β cell survival and insulin secretion, like DNA methylation, chromatin architectural modification, and non-coding RNA (Kowluru and Mishra, 2017). The application of CRISPR/Cas9 technology on the targeted epigenome would be able to differentiate cell types to improve insulin production (Dinić et al., 2022). These findings underscore OS as a central mediator of β-cell failure in diabetes, highlighting the need for targeted antioxidant strategies (see Figure 3) that mitigate ROS without disrupting physiological redox signaling (Zaher and Stephens, 2025).

### 6.2 Hyperglycemia-induced oxidative stress pathways

Glycated hemoglobin (HbA1c), which averages blood sugar levels over the previous two to 3 months within total red blood cell hemoglobin, is a measure of the degree of glucose’s natural binding to hemoglobin (glycation). In persons without diabetes, it is typically 5.7% or less. The percentage of HbA1c rises when blood sugar levels increase over time. Elevated HbA1c promotes the intraerythrocytic generation of highly reactive free radicals (AGEs), which alter membrane functions and those of different cellular systems by binding to RAGE and producing cytokines and intracellular glyco-OS (Nuñez-Musa, 2024). By causing hyper-aggregation and hyper-viscosity, which ultimately lead to inflammation and atherogenic events, these results exacerbate endothelial damage (Nabi et al., 2019). Furthermore, changes in the metabolism and biodisposition of intracellular iron are brought about by the oxidative phenomena, resulting in the production of ferryl-hemoglobin. This more unstable substance subsequently triggers conversion processes into hemoglobin multimers. These multimers promote vascular intima injury, which increases endothelial permeability, monocyte adhesion, and macrophage accumulation, leading to plaque development (Bozza and Jeney, 2020). In general terms, the pathophysiological cascade that arises from non-enzymatic protein glycosylation linked to persistent hyperglycemia determines the substrate for the cardiovascular effects of DM.

The polyol pathway consumes up to 30% of intracellular glucose under hyperglycemic conditions, with aldose reductase converting glucose to sorbitol in an NADPH-dependent reaction that depletes this critical cofactor required for glutathione reductase activity (Gupta, 2024). This glutathione depletion impairs cellular antioxidant capacity, leaving cells vulnerable to ROS accumulation. The formation of AGEs increases 3-5-fold in diabetes, as glucose-derived carbonyl groups nonenzymatically modify proteins and lipids; these AGEs then bind to RAGE receptors, activating NOX enzymes and generating O_2_•^-^ while triggering pro-inflammatory NF-κB signaling (Yan et al., 2023). The PKC pathway becomes activated through hyperglycemia-induced increases in diacylglycerol (DAG), with PKC-β and PKC-δ isoforms particularly implicated in stimulating NOX activity while inhibiting AMPK-mediated antioxidant responses (Geraldes and King, 2018). Finally, excessive glucose flux through the hexosamine pathway leads to UDP-GlcNAc accumulation and subsequent O-GlcNAcylation of serine/threonine residues on antioxidant enzymes, like SOD, reducing their activity (Paneque et al., 2023). These pathways converge to create a state of chronic OS that further exacerbates insulin resistance and β-cell dysfunction, and plasma F2-isoprostanes (markers of lipid peroxidation) correlate strongly with HbA1c levels (Tang et al., 2018). This oxidative milieu persists even after glucose normalization, contributing to the phenomenon of “metabolic memory” observed in clinical trials, where early intensive glycemic control provides long-term protection against complications (Yang et al., 2024). Recent studies using isotopic tracer techniques have quantified the relative contributions of these pathways, revealing that mitochondrial O_2_•^−^ production initiates about 45% of hyperglycemia-induced damage, while PKC activation and AGE formation account for 30% and 25%, respectively (Chronic Complications of Diabetes Mellitus, 2024; Pardina et al., 2024). This quantitative understanding is now driving the development of pathway-specific inhibitors, such as PKC-β selective antagonists, SGLT2 inhibitors, and glyoxalase-1, which have shown promise in preclinical models for breaking this cycle without globally suppressing physiologically important ROS signaling -Figure 3 (Oliveira et al., 2023; Klen and Dolžan, 2023; Xing et al., 2024).

### 6.3 Oxidative stress and insulin resistance

The Insulin Resistance (IR) explains the failure of muscles, fat, and liver to respond well to insulin and their incapacity to take up glucose from the blood. To compensate, the pancreas increases insulin production, but cannot achieve positive results, and a progressive disorder of lipid and glucose metabolism takes place. The IR usually progresses in silence and manifests clinically years after being present in the form of hyperglycemia, favoring atherogenesis and plaque progression in a curvilinear relationship with vascular disease, even in the absence of hyperglycemia (Beverly and Budoff, 2020). OS is a critical link between metabolic overload and IR through multiple interconnected mechanisms that impair insulin signaling at various levels. At the cellular level, ROS directly modify key components of the insulin signaling cascade, including insulin receptor substrate (IRS) proteins and the insulin receptor itself. H_2_O_2_ and O_2_•^−^ activate stress-sensitive serine/threonine kinases such as JNK, IKKβ, and PKCθ, which phosphorylate IRS-1 at inhibitory serine residues (Ser307, Ser612), disrupting its interaction with the insulin receptor and promoting proteasomal degradation (Roden et al., 2017). Lipid peroxidation products like 4-HNE form covalent adducts with cysteine residues in the insulin receptor β-subunit, reducing its tyrosine kinase activity by up to 60% (Furukawa et al., 2017). Mitochondrial dysfunction in skeletal muscle, characterized by reduced ATP synthesis rates and increased electron leak, generates excessive ROS that activates PKCε, leading to impaired insulin-stimulated GLUT4 translocation (Regina and Tong, 2025). Adipose tissue contributes to systemic OS through NOX4 activation and reduces adiponectin secretion, creating a pro-inflammatory milieu that further exacerbates insulin resistance (Den Hartigh et al., 2017). OS also induces ER stress, activating the UPR and its downstream effector XBP-1, which suppresses insulin receptor expression (Zhang et al., 2025).

Sustained elevated glucose levels favor an imbalance between the availability of endothelial NO and the accumulation of ROS. This imbalance triggers a chain of pathophysiological events that lead to enzymatic dysfunction in endothelial homeostasis, inflammation, and cell proliferation. More particularly, a strong activation of PKC in the endothelium of diabetic patients is observed in the increased generation of ROS and microvascular OS (Nuñez-Musa, 2024), and vasoconstriction potentiates and insulin signaling peripherally impairs (Robson et al., 2018). With greater and more extensive experiences, PKC is a possible therapeutic target for the treatment of vascular complications in diabetics. Inflammatory factors, in addition to OS, play a significant role in the development of diabetes by promoting IR through the alteration of β-cell function and interference with insulin signaling (Oguntibeju, 2019). Given the crucial role of interleukins in mediating inflammation through both pro- and anti-inflammatory properties, various interleukins have been investigated in DM2 patients (Alfadul et al., 2022; Yousef et al., 2023). OS and inflammation contribute to endothelial dysfunction through the inactivation of NO, which increases the risk of DM2, primarily through the renin-angiotensin-aldosterone system (Martyniak et al., 2024). These findings highlight OS and inflammation as both a cause and consequence of IR, creating a cycle that can be interrupted by targeted antioxidant approaches and/or lifestyle interventions that enhance endogenous antioxidant capacity (see Figure 3).

### 6.4 Oxidative stress and diabetic vascular complications

OS plays a central role in the pathogenesis of diabetic vascular complications through multiple interconnected pathways that promote endothelial dysfunction, vascular inflammation, and extracellular matrix remodeling. Chronic hyperglycemia-induced ROS overproduction, primarily derived from mitochondrial ETC leakage and NOX activation, leads to eNOS uncoupling due to BH4 oxidation, reducing NO bioavailability and impairing vasodilation (Giacco and Brownlee, 2010). Simultaneously, ROS activate PKC isoforms and the AGE-RAGE signaling cascade, which upregulate pro-inflammatory cytokines (TNF-α, IL-6) and adhesion molecules (VCAM-1, ICAM-1) through NF-κB activation (González et al., 2023). In the macro-vasculature, OS accelerates atherosclerosis by promoting LDL oxidation, macrophage foam cell formation, and plaque instability via matrix metalloproteinase (MMP) activation (Caturano et al., 2025). Microvascular complications are equally driven by oxidative damage i.e., in diabetic retinopathy, ROS-induced VEGF overexpression through HIF-1α stabilization leads to pathological angiogenesis (Kowluru and Mishra, 2017), while in diabetic nephropathy, podocyte injury results from NOX4-derived O_2_•^−^ and mitochondrial ROS-mediated apoptosis (Jha et al., 2024). Clinical studies demonstrate strong correlations between OS markers and vascular complications including diabetic kidney disease (Forbes and Thorburn, 2018; Caturano et al., 2025).

Furthermore, due to a decrease in their destruction rate and/or a reduction in the production of CAT, SOD, and GPx, excess ROS promotes the deleterious changes associated with OS. Ultimately, tissues are more vulnerable to OS and, consequently, to diabetic complications, especially vascular problems, which are the main cause of comorbidity and mortality in DM2. Several challenges need to be considered while creating customized antioxidant treatments. These include gene therapy based on Mn-SOD used for systemic or organ-specific applications to protect tissues from OS-related damage (Greenberger et al., 2021) and antioxidant enzyme gene transfer, which has been investigated as a potential method to deliver antioxidant enzymes (Lombardi et al., 2017; Anwar et al., 2024).

Emerging therapeutic strategies aim to target these pathways specifically like MATs, NOX inhibitors, and Nrf2 activators, though clinical success has been limited by off-target effects and the dual role of ROS in physiological signaling (Pergola et al., 2021). New strategies include nanoparticle-based antioxidant delivery to vascular tissues and personalized redox therapies guided by genetic and biomarker profiling (Tang et al., 2024). These advances emphasize the necessity for precise interventions in time and space that reduce harmful oxidative stress while maintaining beneficial redox signaling in diabetic blood vessels (Figure 3).

Pant et al. (2023) have reviewed the present status of long non-coding (lnc) RNA as a diagnostic biomarker and potential regulator of OS in vascular complications of DM2 and the recent advances in using MTAs in different animal models and clinical trials. They found that the expression level of lncRNA has shown a strong correlation with OS, indicating that they might modulate redox status in diabetic complications, and the emerging scientific evidence depicts that MTAs can mitigate mitochondrial OS caused by hyperglycemia in DM, providing promising OS-targeting therapeutic strategies. However, most studies to date investigating the effect of MTAs on OS have been conducted using animal models or cell cultures rather than diabetic patients. The epigenetic role in this context is not fully understood. Still, changes in DNA have been identified that could be meaningful in the identification and design of the next therapeutic strategies. It is considered that mitochondrial mutations can accumulate over time. Difficulties in this field arise due to the particularities encountered at the level of each individual. Large and complex studies are needed to identify and detail the changes at the mitochondrial level and the therapeutic approaches (Cojocaru et al., 2023).

Sodium-glucose cotransporter 2 inhibitors (SGLT2i) have demonstrated pleiotropic effects, including reducing mitochondrial ROS generation. In older patients with DM2 and HTN, an observational study correlated empagliflozin administration with decreased endothelial fragility and OS markers, attributable to reduced mitochondrial ROS production in endothelial cells (Mone et al., 2022). A 6-month controlled trial in diabetics with ischemic heart disease showed that empagliflozin attenuated both inflammation and systemic oxidative stress, resulting in clinically significant cardiovascular benefits (Gohari et al., 2022). Preclinical studies revealed that dapagliflozin activates the SIRT6–FOXO3 pathway to reduce myocardial fibrosis and oxidative damage in diabetic cardiomyopathy models (Ma et al., 2024). The use of compounds that stimulate Sirtuin and AMPK modulators SIRT1/SIRT6 (e.g., resveratrol, NMN) has shown potential to restore mitochondrial biogenesis and attenuate OS in insulin-resistant tissues (Wang et al., 2023), although robust clinical trials are lacking. Additionally, specific-purpose antioxidants introduced as potential treatment coadjutants, SOD (EUK-134) and CAT (manganese porphyrins) mimics demonstrated efficacy in animal models of diabetic nephropathy and resistant HTN opening the door to future human trials (Wilcox and Aslam, 2021).

### 6.5 Oxidative stress and diabetic neuropathies

Diabetic neuropathies encompass a heterogeneous group of disorders affecting multiple organ systems, all sharing OS as a common pathogenic mediator. These complications can be broadly categorized into microvascular, macrovascular, and neural manifestations. Each has distinct clinical features but shares overlapping molecular mechanisms driven by chronic hyperglycemia-induced ROS overproduction. The most prevalent form, Distal Symmetric Polyneuropathy (DSPN), results from oxidative damage to Schwann cells and sensory neurons in peripheral nerves (Panou et al., 2025). Mitochondrial O_2_•^−^ overproduction in dorsal root ganglia activates multiple injurious pathways, like the polyol pathway flux, which depletes NADPH, reducing glutathione regeneration (Mapanga and Essop, 2016); the AGE-RAGE signaling, which induces axonal atrophy through p38 MAPK activation (Sango et al., 2025), and the PKC-β activation, which impairs nerve blood flow via endothelin-1 overexpression (Chong and Souayah, 2025). Clinically, this manifests as progressive sensory loss (≥5.07 g monofilament testing) and neuropathic pain (DN4 score ≥4), correlating with serum 8-OHdG levels (Asim et al., 2024).

Hyperglycemia-induced mitochondrial dysfunction in neuronal and Schwann cells leads to excessive O_2_•^−^ production at complex I and III of the ETC, which initiates a cascade of injury through four primary mechanisms (Wu L. et al., 2024). First, ROS directly modifies ion channels, oxidizing sulfhydryl groups on voltage-gated Na+ channels, resulting in abnormal action potential propagation and contributing to the paradoxical coexistence of pain and numbness (Elafros et al., 2022). Second, OS activates the polyol pathway, depleting NADPH reserves and reducing glutathione levels, leaving nerves vulnerable to lipid peroxidation—a process evidenced by 3-fold higher levels of 4-HNE in sural nerve biopsies from diabetic patients with neuropathy compared to controls (Chong and Souayah, 2025). Third, ROS promotes microvascular insufficiency by oxidizing BH4, leading to eNOS uncoupling and reduced endoneurial blood flow, with clinical studies showing a strong inverse correlation between skin biopsy-measured intraepidermal nerve fiber density and serum 8-OHdG levels (Carnicer et al., 2021). Fourth, OS triggers inflammatory cascades through NF-κB-mediated upregulation of TNF-α and IL-6, which further damage nerve fibers via Wallerian degeneration (Pop-Busui et al., 2016).

Cardiovascular Autonomic Neuropathy (CAN) is the most lethal form, involving oxidative damage to parasympathetic ganglia and sympathetic nerve terminals. In CAN, mROS in the sinoatrial node reduce heart rate variability, and NOX2-derived O_2_•^−^ in vascular smooth muscle causes orthostatic hypotension (Singh et al., 2024). Late-stage CAN (Ewing score >7) carries a 5-year mortality of 50%, linked to plasma F2-isoprostanes >450 pg/mL (Pop-Busui et al., 2022). This classification underscores the need for phenotype-specific antioxidant approaches in diabetic neuropathies, moving beyond “one-size-fits-all” therapies. Future trials should incorporate redox biomarker stratification and advanced neuroimaging to match mechanisms with interventions (see Figure 3).

New therapeutic strategies focus on MATs approaches, including MitoQ, Mito-TEMPO, and nano-Mito-PBN (Pang et al., 2025; Pant et al., 2023; Wu et al., 2019); Nrf2 activators to reduce neuropathic pain, like sulforaphane (Aranda-Rivera et al., 2024), and PKC-β inhibitors, which preserved intraepidermal nerve fibers in clinical studies, like ruboxistaurin (Rendell, 2021). Mito-apocynin (Mito-Apo), a mitochondria-targeted derivative of apocynin, concentrates at the inner mitochondrial membrane to inhibit NOX2 directly, where ROS production is highest. In a lipopolysaccharide (LPS)–induced endotoxemia mouse model, oral Mito-Apo markedly reduced systemic oxidative markers (e.g., 4-HNE) and preserved mitochondrial function, while standard apocynin showed minimal efficacy (Padhi et al., 2019). Another study has demonstrated that diapocynin exhibits a lower IC_50_ for NOX2 inhibition (sub-micromolar) compared to parent apocynin, effectively blocking p47^phox^ translocation and attenuating endothelial superoxide generation in isolated vessel assays (Juric et al., 2023). In diabetic nephropathy models, GKT137831 reduced glomerular ROS (measured by dihydroethidium staining), decreased albuminuria by over 50%, and attenuated mesangial expansion—effects that correlated with diminished NOX4 expression in podocytes. Additionally, in bleomycin-challenged rodent lungs, setanaxib treatment cut lung hydroxyproline content by ∼40%, suppressed peroxynitrite formation (assessed via EPR spin trapping), and improved compliance, demonstrating its capacity to interrupt NOX-driven fibrogenesis (Thannickal et al., 2023). Collectively, these results highlight the ability of highly engineered apocynin derivatives to specifically inhibit the NOX2 isoform at its mitochondrial source, while substances such as GKT137831 support the more general NOX inhibition strategy to reduce vascular and tissue-specific ROS in fibrotic diseases and diabetic nephropathy.

Recent advances in redox proteomics have identified novel biomarkers like peroxiredoxin-3 oxidation that predict neuropathy progression 5 years before clinical onset (Bourgonje et al., 2024), while nanoparticle-based delivery systems are being tested to selectively target antioxidants to damaged nerves without systemic side effects (De et al., 2025). These developments demonstrate the central role of OS in diabetic neuropathies and highlight promising avenues for mechanism-based therapies that could finally break the cycle of nerve injury in diabetes.

## 7 Clinical trial protocol design for antioxidant therapy in hypertension and diabetes

The selection of appropriate OS biomarkers is critical for assessing the efficacy of antioxidant therapies employed in the treatment of HTN and DM from the results of clinical trials. These biomarkers yield valuable insights regarding i) products of lipid peroxidation, ii) products of protein modification, iii) activities of antioxidant enzymes, and iv) DNA modifications that may influence gene expression (Mañon-Rossi et al., 2016). A significant portion of the clinical research concerning the application of antioxidants in HTN and DM has not fully incorporated information about OS biomarkers (Sies, 2020). Marrocco et al. (2017) proposed that biases resulting from independently applied methodologies could be alleviated by the utilization of OS indices that encompass multiple markers pertinent to the objectives of the clinical study and its implications for clinical practice.

Experimental evidence indicates that an accurate diagnosis of OS can enhance the efficacy of antioxidant therapies in patients suffering from HTN and DM. The Oxidative Stress Index (OSI) (Nuñez-Sellés et al., 2019) offers valuable support within a rigorously designed clinical protocol aimed at optimizing the administration of antioxidant treatments. This methodology endeavors to augment the effectiveness of therapies while minimizing comorbidities when coupled with standard treatments currently utilized for these conditions. On the other hand, it is essential to focus on clinical trial endpoints related to disease progression and improvement in antioxidant studies, as these are more meaningful than simply measuring variations in overall survival OS biomarkers or patient mortality. When designing antioxidant clinical trials in HTN and DM, it is important to demonstrate how antioxidant therapy (preferably used as an adjunct to standard treatment) can lead to improvements in BP for HTN and HbA1c levels for DM. The increasing trend of employing antioxidants in conjunction with standardized antihypertensive or antidiabetic medications appears to represent one of the most effective strategies for improving the prognosis of HTN and DM. This effectiveness must be correlated to the capacity to manage the molecular mechanisms underlying vascular function, metabolic equilibrium, and redox states through the intentional management of specific antioxidants (Sorriento et al., 2018). Additionally, implementing antioxidant therapies may play a pivotal role in addressing complications or deterioration stemming from physiological imbalances associated with AHT and DM.

Designing robust clinical trials to evaluate antioxidant therapies in HTN and DM requires addressing unique challenges, including patient heterogeneity, biomarker variability, and the dual role of OS as both cause and consequence of disease progression. An optimal protocol should incorporate stratified randomization based on OS biomarker profiles (e.g., plasma MDA, urinary 8-OHdG) to identify subgroups most likely to benefit. Control and/or placebo groups must be integrated according to the ethnic composition of the target population. One example in diabetic cohorts treated with MitoQ, the clinical protocol was designed combining DM endpoints (HbA1c and endothelial function) in a more preferable way than isolating redox-specific effects from glucose-lowering biomarkers. MitoQ improved flow-mediated dilation (FMD) by 3.1% in DM2 patients independent of glycemic changes (Carlini et al., 2024). Another study combined BP values with F2-isoprostanes plasma values as trial endpoints, leading to a correlation of these OS biomarkers with a BP reduction with sulforaphane as compared to the placebo group (Bai et al., 2015).

Dose-finding phases are critical, given the U-shaped efficacy curve of antioxidants. For example, a high dose of N-acetylcysteine (1,200 mg/day) paradoxically increased oxidative damage in hypertensive patients, while 600 mg/day reduced MDA by 27% (Szkudlinska et al., 2016). To mitigate placebo effects, crossover designs with washout periods (e.g., 4 weeks) are advantageous. Another example is flavonoid glycoside (rutin) administration to DM2 patients has improved BP, the levels of antioxidant enzymes, and quality of life (Bazyar et al., 2023), but the sample size (n = 19) acts as a handicap for its significance. The use of antioxidant phytochemicals has also been claimed to have potential benefits (Akpoveso et al., 2023; Arabshomali et al., 2023), but again, randomized clinical trials are scarce and contradictory. The low bioavailability and absorption of antioxidant phytochemicals currently limit their utility in the clinic, and further studies are needed to explore the antidiabetic potentials of phytochemicals, improve their absorption and bioavailability, and explore their potential side effects in clinical trials.

Okoduwa et al. (2015) investigated the correlation between age-related changes and the antioxidant defense system in patients with HTN and/or DM2. The cohort consisted of 200 patients divided into four groups, each containing 50 subjects: a diabetic group, a hypertensive group, a hypertensive-diabetic group, and a control group of non-diabetic, normotensive individuals. The results indicated that several OS biomarkers increased with age. Specifically, lipid peroxidation potential rose by 90%, antioxidant enzyme levels increased by more than 10%, and serum selenium levels rose by over 50%. Based on these findings, it was recommended that elderly patients with HTN and DM2 should receive antioxidant supplementation.

Advanced monitoring techniques—such as electron paramagnetic resonance (EPR) for real-time ROS detection and mitochondrial DNA copy number assays—can enhance endpoint precision, as piloted in the MITO-HTN study (Rossman et al., 2018). Finally, long-term safety follow-up (≥12 months) is essential to assess risks of chronic Nrf2 activation (e.g., cancer progression) or antioxidant interference with physiological ROS signaling, highlighted by the bardoxolone methyl experience in diabetic kidney disease (Nangaku et al., 2020). Future trials should prioritize adaptive designs to evaluate combinations (e.g., NOX inhibitors + Nrf2 activators) and digital health tools (e.g., continuous glucose/BP monitoring) for dynamic OS assessment.

The application of antioxidant therapies aimed at lowering AGE could be a helpful tool because high AGE concentrations harm vascular smooth muscle and adversely affect NO function, which in turn impairs endothelial relaxation and prevents NO action against plaque formation from the oxidized form of LDL (Saleh, 2015). According to Csiha et al. (2025), alpha-lipoic acid reduced AGEs in diabetic neuropathy cases, suggesting that it may enhance endothelial function. Antioxidant treatments in patients with DM2 have yet to demonstrate beneficial results, primarily due to the insufficient understanding of the relationship between antioxidant functions and disease progression. One specific target regarding OS and DM2 is Nrf2, which regulates a range of detoxifying genes involved in the response to ROS increase (La Rosa et al., 2020. When Nrf2 is activated, it triggers the transcription of genes that detoxify reactive oxygen species (ROS) and eliminate damaged cellular components. Effective management of blood glucose levels in individuals with type 2 diabetes (DM2) involves a comprehensive approach. A study investigated the blood concentrations of various antioxidant defense biomarkers in a group of 80 patients with DM2 to evaluate the potential correlation between antioxidant defense, glycemic control, and the presence of diabetic complications (Gawlik et al., 2016). The researchers found that Ferric Reducing Ability of Plasma (FRAP), Glutathione Reductase (GSH), and gamma glutamyl transferase (GGT) levels were significantly higher in DM2 patients compared to the control group, while Glutathione Peroxidase (GPx) levels were significantly lower. Additionally, fasting serum glucose showed a correlation with GSH, GPx, FRAP, and GGT, whereas glycosylated hemoglobin (HbA1c) only correlated with GGT. No differences were observed in antioxidant defense biomarkers in patients with chronic diabetes complications and those without. The authors concluded that the overall antioxidant defense system in DM2 is unable to counteract the increased production of ROS. Therefore, they suggested that antioxidant supplementation should be considered as an adjuvant therapy in DM2 treatment. Interestingly, our results about OS biomarkers in DM2 and type 1 diabetes mellitus (DM1) showed that antioxidant therapy should not be recommended in DM1 (Nuñez-Sellés et al., 2019).

## 8 Future directions

Conventional antioxidants face limitations in efficacy and specificity, but emerging strategies—including mitochondria-targeted compounds and Nrf2 activators—address these challenges through three transformative mechanisms:

• Precision targeting

Unlike broad-acting scavengers, these agents accumulate within mitochondria, the primary source of ROS. This subcellular localization enables the direct protection of mitochondrial lipids, proteins, and mtDNA from oxidative damage, a critical advantage in diseases like neurodegeneration and metabolic disorders.

• Endogenous defense amplification

Rather than transient ROS neutralization, these therapies enhance the cell’s innate antioxidant capacity by upregulating glutathione synthesis, antioxidant enzymes, and phase II detoxification systems. This creates a sustained, adaptive response to OS.

• Multipathway disease modulation

By activating Nrf2 and interconnected pathways, they concurrently suppress pro-inflammatory signaling (e.g., NF-κB), reduce apoptotic susceptibility, and improve metabolic efficiency via PGC-1α regulation.

This dual approach-localized ROS interception coupled with systemic redox resilience, offers unparalleled promise for treating chronic conditions (e.g., diabetic complications, cardiovascular disease) where OS drives pathology, thus providing an advantageous therapeutic potential.

### 8.1 Hypertension

The development of dynamic, tissue-specific OS biomarkers represents a critical frontier in HTN management. Current limitations of static plasma/serum measurements (e.g., MDA, F2-isoprostanes) could be overcome through several innovative approaches. Real-time redox monitoring using implantable graphene-based electrochemical sensors is being developed for continuous measurement of vascular H2O2 and O2•− levels. Early prototypes have demonstrated 90% accuracy in correlating BP with endothelial dysfunction in animal models (Zhang et al., 2023). These could be paired with existing Continuous Glucose Monitoring-style wearable technology for simultaneous glucose and OS tracking in metabolic HTN. Mitochondrial redox fingerprinting with advanced mass spectrometry and nuclear magnetic resonance techniques is enabling the identification and quantification of OS biomarkers simultaneously at very low concentrations (Petrova and Guler, 2025). In this sense, important advances have been reported to define the mitochondrial profile metabolites in OS situations, such as cancer. These methods combined allow for the simultaneous identification and imaging of various free radicals, providing metabolic and biochemical data revealing significant differences between whole-cell and mitochondrial metabolite profiles (Domingo-Ortí et al., 2023; Krishnamurthy et al., 2024). This approach provides insights into mitochondrial metabolic alterations, opening a gate to targeted therapeutic actions. Over 200 distinct mitochondrial redox compounds have been identified from a single blood draw for fingerprinting (Chen et al., 2019). Another study identified a distinct 12-metabolite signature that predicted progression to resistant HTN with 82% accuracy (Al-Ashmar et al., 2024).

Redoxomics is a novel type of omics based on knowledge about the role of redox homeostasis for the healthy state of cells and their pathogenesis in several diseases (Nakhod et al., 2024). The need of redox-sensitive fluorescent tags (e.g., roGFP2) coupled with microfluidic cell sorting will allow the profiling of OS heterogeneity within endothelial, vascular smooth muscle, and immune cell populations (Schwarzländer et al., 2016).

The next-generation of antioxidant interventions will focus on spatial and temporal precision, like the NOX isoform-specific nanotherapy based on lipid nanoparticles encapsulating NOX1 siRNA (Cartaya et al., 2019). Experimental results in primate models achieved a 70% knockdown in vascular smooth muscle cells without hepatic toxicity, and a first-in-human trial is planned to start in 2025.

Chronotherapeutic Nrf2 activation is being studied as an alternative for HTN treatment (Serrano and Medina, 2025). Some trials have reported that Nrf2 activators, such as sulforaphane, bardoxolone methyl, and dimethyl fumarate, have demonstrated promise in lowering OS and enhancing endothelial function thus far (Wu Q. et al., 2024). The majority of these studies, whether conducted on humans or animals, aimed to improve effectiveness by matching diurnal BP peaks as possible supplementary resources for antihypertensive medications (Hermida et al., 2016; Tanase et al., 2022). The Nrf2 transcription factor, which aids in OS defense, is regulated by the circadian rhythm (Pekovic-Vaughan et al., 2014). This control is crucial for avoiding fibrosis and tissue damage, and it might be relevant to the development of HTN.

Mitochondrial protonophore therapy is considered an attractive way of HTN treatment (Myszko et al., 2025). Since mitochondrial failure appears to be a major factor in the development of target organ damage linked to HTN, mitochondria have emerged as a possible target for treatment. For several years, researchers have been investigating the use of mitochondrial uncouplers, such as protonophores and UCP2, to modify mitochondrial activity to achieve modulatory action on this organelle. These uncouplers may lower ROS because they increase energy expenditure by moving protons across the inner mitochondrial membrane (Minanimo-Muta et al., 2018; Nesci and Rubattu, 2024). Controlled uncoupling with modified dinitrophenol analogs (e.g., DNP-Mito) reduced mtROS by 65% while maintaining ATP synthesis in hypertensive rat models (Kotova and Antonenko, 2022).

Experimental gene therapies delivering antioxidant enzyme genes (e.g., CAT, SOD, GPx) via viral vectors or nanoparticle carriers have shown promise in animal models by bolstering intrinsic defenses (García-González et al., 2024; Padmanaban et al., 2023). Some studies demonstrate the potential of nanoparticle-mediated delivery of antioxidant enzymes to protect neurons from OS. CAT was encapsulated within poly-lactic-co-glycolic acid (PLGA) nanoparticles, which retained approximately 99% of the enzyme’s activity. These nanoparticles provided sustained release of CAT over a month and effectively protected human neurons from H_2_O_2_-induced oxidative damage, preserving neuronal morphology and function (Ashok et al., 2022). A recent study developed human serum albumin (HSA)-based nanoparticles stabilized with polyethyleneimine (PEI) to co-deliver the SOD 1 gene and sulforaphane, a known Nrf2 activator. The nanocarriers demonstrated high biocompatibility and transfection efficiency (∼66%) in human lung epithelial cells (L-132). The combined delivery system effectively enhanced antioxidant defenses, suggesting potential for treating diseases associated with oxidative stress (Naqvi et al., 2022).

Advancing OS Therapies in HTN should consider several alternatives. First, the optimization of the Nrf2 pathway through targeted drug delivery, e.g., lipid nanoparticles (LPNs) loaded with sulforaphane or bardoxolone methyl could enhance endothelial uptake while avoiding systemic side effects (Zhang et al., 2024), and LNPs conjugated to VCAM-1 antibodies for inflamed vasculature targeting (Gu et al., 2025). Translating all these advances mentioned above to clinical practice will require the construction of redox phenotyping panels with several biomarkers incorporating various sources of biomolecular and clinical data to guarantee a higher robustness and power of separation for a clinical test (Smith et al., 2021). A 10-parameter panel, including not only classical antioxidant tests (FRAC, Trolox, DPPH) but also endogenous antioxidant enzymes and protein degradation, has shown its advantages in stratifying OS status in different HTN degrees (Nuñez-Sellés et al., 2019). The development of AI-driven treatment algorithms through supervised machine learning is successfully automating the process of evaluation and quantification of oxidative damage in biological samples, as well as extracting useful data from the abundance of experimental results (Pantic et al., 2022). Machine learning models may integrate redox biomarkers, pulse wave analysis, and gut microbiome data to predict optimal antioxidant regimens with high accuracy. Recently, microbiome redox modulation has appeared in the scenario. (Million and Raoult, 2018). Gut microbiota dysbiosis contributes to HTN through mechanisms involving OS and inflammation (Li et al., 2021). “Gut redox” focuses on the intricate redox equilibrium within the gastrointestinal tract, encompassing a dynamic interplay of ROS and antioxidants (Prasad et al., 2024). Akkermansia muciniphila supplementation has been shown to reduce BP through the reduction of OS markers, likely through increased propionate production, but its translation to the clinic is quite controversial (Lakshmanan et al., 2022). The impact of microbial metabolites on vascular function and its potential of targeting gut microbiota to modulate redox balance and BP is a promissory gate to bettering HTN syndrome (Li et al., 2021). Interventions targeting gut microbiota, such as probiotics and dietary modifications, show promise in managing OS and blood pressure.

These future directions collectively represent a transformative shift from reactive BP management to proactive redox-based prevention and personalized treatment of hypertensive vascular disease. The integration of advanced biomarkers, targeted therapies, and systems biology approaches promises to revolutionize our understanding and management of OS in HTN.

### 8.2 Diabetes

The growing recognition of OS as both a cause and consequence of diabetes pathogenesis has fundamentally reshaped the understanding of this metabolic disorder. By replicating glucose consumption and changes in oxidative phosphorylation, hyperglycemia and hyperlipidemia exacerbate inflammation. Cell migration causes the inflammatory response to expand into the vasculature, islets, and adipose tissue. Due in large part to endothelial damage and oxidative damage to the endoplasmic reticulum, glycolipotoxicity comprises the primary cause of the inflammatory response triggered in DM2 and ultimately resulting in IR. These arguments, which center on the molecular effects of OS precursors or modulators, imply that certain anti-inflammatory products might be taken into consideration as part of the antidiabetic treatment, just as they have been for myocardial infarction (Nuñez-Musa, 2024).

Mounting evidence positions redox imbalance not merely as a secondary phenomenon but as a primary driver of the disease continuum - from initial β-cell dysfunction to devastating micro- and macro-vascular complications. This paradigm shift has unveiled critical limitations in current antioxidant approaches while simultaneously opening unprecedented opportunities for mechanism-targeted interventions. The future of antioxidant therapy in DM lies in overcoming the limitations of broad-spectrum approaches by developing targeted, personalized, and mechanism-based interventions that address the nuanced role of OS in disease progression.

Next-generation interventions will focus on spatial and temporal precision protocols. β-Cell-specific redox modulators, as the conjugated glucagon-like peptide-1 (MitoTEMPO) bv nanoparticles have achieved 80% β-cell targeting efficiency in primates, preserving insulin secretion under glucotoxic conditions (Marques et al., 2024). Dual NOX/SGLT2 inhibitors as the hybrid compound GKT137831 (inhibitor of NOX1 and NOX4) and empagliflozin (SGLT2 inhibitor) have shown synergistic effects, improving insulin sensitivity by 40% in diabetic rodent models (Karakasis et al., 2025). Additionally, cutting-edge metabolomic approaches are revolutionizing the ability to quantify mitochondrial oxidative stress in diabetes.

Emerging strategies focus on mitochondrial-specific antioxidants (e.g., MitoQ, SS-31), which have shown promise in preclinical and early-phase clinical trials by reducing mtROS and improving endothelial function in diabetic patients (Mason et al., 2022). Recent advances in Nrf2 activators, such as synthetic triterpenoids and natural compounds, highlight the need for temporal precision—activating Nrf2 early to preserve β-cell function but avoiding chronic overexpression, which may promote cancer (Mohamadi et al., 2023). Nanotechnology is poised to revolutionize delivery, with ROS-responsive nanoparticles encapsulating antioxidants (e.g., mangiferin, curcumin, resveratrol) for targeted release in pancreatic islets or atherosclerotic plaques (Cheng et al., 2024). Additionally, dual-target therapies that combine OS reduction with conventional antidiabetic effects—such as SGLT2 inhibitors with NOX4 inhibitors—are under investigation to address multifactorial pathology (Balogh et al., 2023). Future clinical trials should integrate deep phenotyping techniques, such as redox proteomics and mitochondrial DNA damage assays, along with AI-driven biomarker panels. This approach will help identify patients most likely to benefit from interventions, advancing beyond just HbA1c levels to include dynamic OS metrics like real-time ROS sensors (Geraskevich et al., 2024). Lastly, lifestyle-integrated approaches, such as time-restricted eating (TRE) combined with low-dose antioxidants, may amplify endogenous defenses while minimizing pharmacological risks (Varady et al., 2022). The path forward requires a paradigm shift from reactive antioxidant supplementation to precision redox medicine, leveraging genomics, microbiome science, and targeted delivery systems to break the gap of OS in DM.

## 9 Conclusion

The management of OS has evolved from nonspecific free-radical scavenging to precision-driven interventions that address both the sources and consequences of redox imbalance. Traditional biomarkers, such as MDA, F_2_-isoprostanes, protein carbonyls, and 8-OHdG, combined with advanced techniques like EPR spectroscopy and multi-omics, now enable a comprehensive assessment of oxidative injury across lipids, proteins, DNA, and metabolites. Therapeutically, small-molecule antioxidants and nutraceuticals laid the groundwork, but their limited organelle specificity and bioavailability have driven the development of mechanism-based approaches. Mitochondria-targeted compounds directly neutralize ROS at its primary source, while Nrf2 activators amplify endogenous defense systems to sustain redox homeostasis. Gene- and enzyme-delivery platforms, ranging from AAV vectors encoding CAT/SOD to nanoparticle nanozymes, further bolster intracellular antioxidant capacity. In cardiometabolic contexts such as DM2 and HTN, agents like SGLT2 inhibitors and AMPK/Sirtuin modulators illustrate how metabolic correction can dovetail with oxidative protection. Finally, isoform-selective NOX inhibitors exemplify targeting of disease-relevant ROS sources. Looking ahead, integrating precise diagnostics with tailored antioxidant therapies promises to transform the treatment of chronic disorders driven by OS, like DM2 and HTN. Continued innovation in delivery technologies, patient stratification, and rigorous clinical validation will be essential to realize the full potential of these next-generation interventions.
